# Bouveret Syndrome: A Rare Case of Gallstone Ileus Further Complicated by Stone Migration

**DOI:** 10.7759/cureus.11219

**Published:** 2020-10-28

**Authors:** Hannah Dunlop, Mohammad R Goodarzi

**Affiliations:** 1 General Surgery, University Hospital Wishaw, Glasgow, GBR

**Keywords:** bouveret's syndrome, gallstone, gastric outlet obstruction, gall bladder diseases

## Abstract

Bouveret syndrome is a rare cause of gastric outlet obstruction due to gallstone impaction in the pylorus or proximal duodenum. This paper reports a case of Bouveret syndrome in a 66-year-old male patient in whom pre-operative investigations revealed a gallstone within the distal stomach, but spontaneous migration of the stone resulted in intraoperative difficulty requiring further surgical exploration than originally anticipated.

## Introduction

Gallstone ileus complicates 0.3-0.5% of cases of cholelithiasis. It classically occurs when a gallstone becomes impacted at the terminal ileum causing small bowel obstruction. An even more rare subtype of gallstone ileus, accounting for only 1-3% of cases, is referred to as “Bouveret syndrome” [1]. The term Bouveret syndrome is used to describe gastric outlet obstruction due to gallstone impaction in the duodenum or pylorus as a result of fistulous communication between the biliary tract and the proximal small bowel or stomach. Although it is named after Leon Bouveret who, in 1896, described two cases of gastric outlet obstruction secondary to a stone retained in the duodenal bulb, the syndrome was originally described by Beaussier in 1770 [2].

Although rare, the great clinical importance of Bouveret syndrome is related to its high mortality rate of up to 27% and furthermore by the fact that morbidity can reach 50% in patients of advanced age and comorbid states or when there is delayed intervention [3]. In addition to its rarity, the clinical challenge of detecting and diagnosing Bouveret syndrome also lies in its vague presentation. Indeed, only 50% of patients presenting with Bouveret syndrome will have a known gallstone disease, and the majority will not present with classical symptoms [4].

This case report highlights the challenges that can be faced in diagnosing Bouveret syndrome and also how gallstone migration resulted in discord between the clinical presentation, radiological findings, endoscopic appearance, and intraoperative findings in a patient presenting with Bouveret syndrome. Therefore, the aim of this case report is not only to increase awareness of Bouveret syndrome and to highlight the difficulties that may be encountered in its diagnosis, but also to acknowledge that stone migration may complicate the clinical scenario and necessitate more extensive surgical management than first anticipated.

## Case presentation

A 66-year-old male patient with a background of duodenal ulcer repair presented to the emergency department with a four-hour history of non-bilious vomiting immediately after oral intake. He also reported a two-day history of constipation. Examination revealed a generally tender and mildly distended abdomen with normal bowel sounds. Admission blood tests and chest and abdominal X-rays were non-specific, with the only significant derangement being a C-reactive protein of 138 mg/L. The main differential diagnoses documented on admission were subacute bowel obstruction or small bowel obstruction secondary to adhesions. He was initially managed with intravenous antibiotics, fluid resuscitation, and analgesia. In addition, a nasogastric tube was placed, which drained 600 mL of dark fluid initially thought to be feculent, over the first 24 hours.

**Figure 1:**
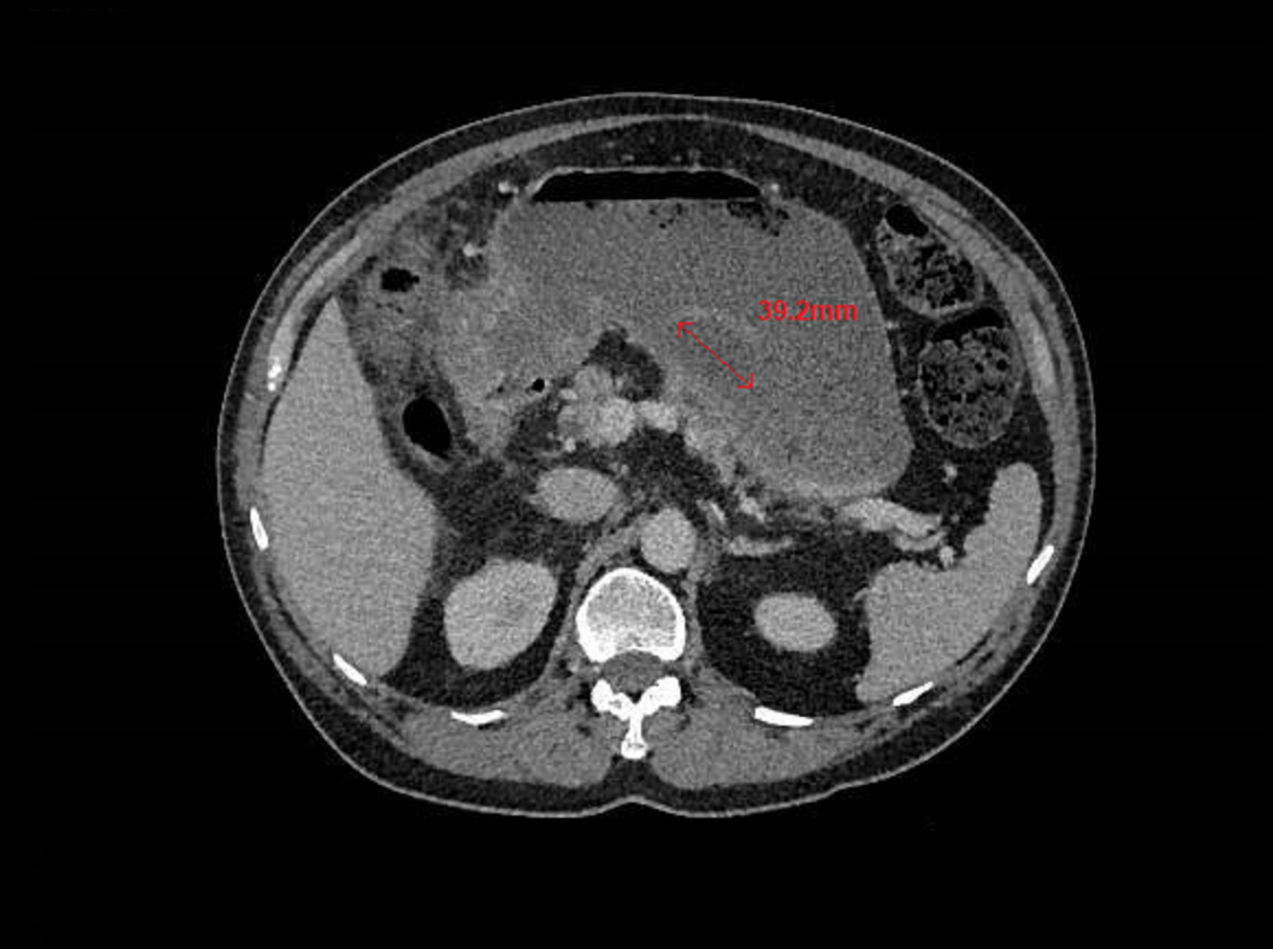
CT demonstrating a 4-cm intraluminal filling defect in the stomach (red arrow)

Computed tomography (CT) of the abdomen and pelvis demonstrated cholecystitis with a fistulous communication between the distal stomach and the gallbladder, as well as a 4-cm intraluminal filling defect within the stomach (Figure 1) of indeterminate nature.

**Figure 2:**
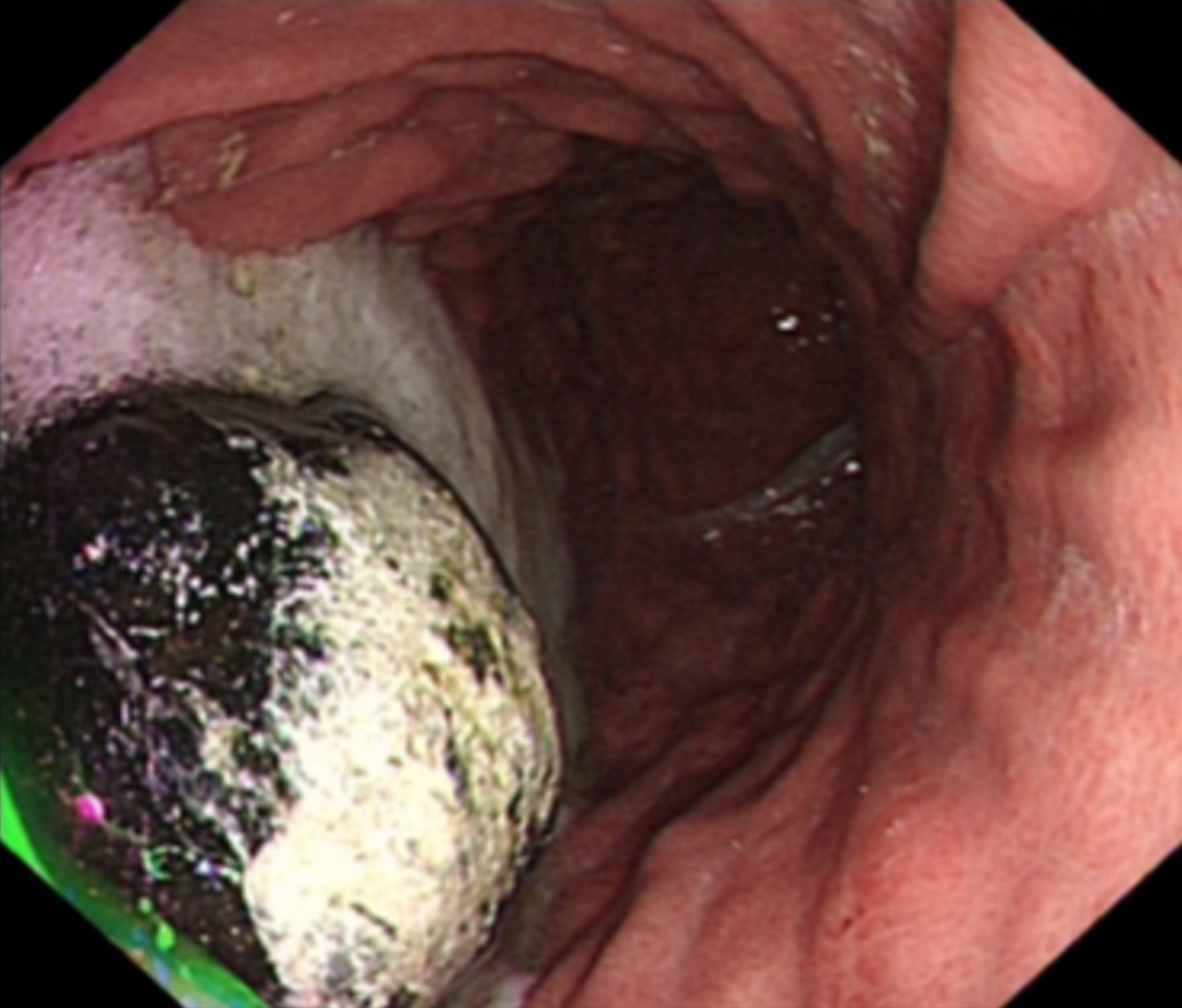
Large gallstone impacted in distal stomach visualized on OGD OGD, oesophago-gastro-duodenoscopy

On oesophago-gastro-duodenoscopy (OGD), the filling defect was identified as a large gallstone (approximately 3-4 cm in diameter) in the distal stomach, but no fistula could be identified in either the stomach or proximal duodenum (Figure 2).

Snare removal of the stone and lithotripsy were attempted at OGD but were unsuccessful as the stone was too large to be grasped by the endoscopic tools available. The patient therefore underwent laparotomy. However, no stone could be felt within the stomach or proximal duodenum. Due to difficulty locating the stone, kocherization and enterotomy were performed, which allowed identification and retrieval of the large stone from a cholecystoduodenal fistulous tract that was then repaired. Post-operatively, the patient was managed in the intensive care unit, made nil by mouth, and administered total parenteral nutrition. Post-operative CT with oral contrast confirmed an intact repair of the fistula. The patient had an uneventful recovery following this and was discharged on post-operative day 9.

## Discussion

Bouveret syndrome is a specific variant of gallstone ileus in which there is proximal enteric obstruction. It classically presents with Mordor's triad, which is defined as a history of gallstones, clinical signs of cholecystitis, and sudden onset bowel obstruction. In the case described in this report, the patient only met one of these criteria, that is, sudden onset bowel obstruction [5]. Interestingly, despite CT-demonstrated cholecystitis, the patient did not have any signs or symptoms in keeping with biliary disease. In addition, plain radiography did not demonstrate bowel obstruction, pneumobilia, or an ectopic gallstone, which are the classical features of gallstone ileus described as Rigler's triad. The lack of classical signs meant that Bouveret syndrome had not been considered as part of the differential diagnosis in this case.

Therefore, the patient underwent further imaging in the form of CT to investigate the cause of his clinical bowel obstruction. This revealed a radio-opaque 4-cm filling defect in the stomach, which was of indeterminate nature. The CT also revealed cholecystitis, which was the first indication of underlying biliary disease. This case highlights the importance of considering Bouveret syndrome early to prevent diagnostic delay in patients who present with sudden onset bowel obstruction even in the absence of other classical features.

The literature suggests that the use of oral contrast with CT may aid diagnosis by not only surrounding the gallstone but also entering the fistulous tract and the gallbladder itself [6]. In this case, subsequent OGD was carried out to characterize the lesion and it was then that the ectopic gallstone was identified, leading to the unexpected diagnosis of Bouveret syndrome. In some cases, endoscopy may be therapeutic if the stone amenable to mechanical, electrohydraulic, or laser lithotripsy [6]. However, in the case described, the stone was too large to be removed with the methods available, including grasp retrieval and attempted lithotripsy.

Apart from the difficulty in initially diagnosing Bouveret syndrome, this case was made even more challenging because of migration of the gallstone and the inability to accurately delineate the anatomy of the fistulous tract pre-operatively. In the presented case, pre-operative investigations (both OGD and CT) visualized the stone within the stomach. However, intra-operatively, the stone was not palpable in either the stomach or the proximal duodenum. Further exploration revealed a cholecystoduodenal fistula with the stone located within its common cavity. This suggests that the stone originally entered the proximal duodenum and then moved in a retrograde fashion into the stomach. It is therefore likely that as it passed back and forth, it impacted distally and intermittently obstructed the gastric outlet. The type of fistula responsible for the communication can vary. In this case, the patient was found to have a cholecystoduodenal fistula; however, CT suggested a cholecystogastric fistula, and no fistula could be identified at OGD. This demonstrates that there may be a requirement for thorough operative exploration in order to correctly identify and repair the fistula in Bouveret syndrome as the true intraoperative findings may not correlate with the pre-operative investigations.

## Conclusions

In conclusion, Bouveret syndrome is a rare diagnosis that poses difficulties in both making the initial diagnosis and in its management. Maintaining a high index of suspicion is key to making early diagnosis and prompt intervention in order to reduce morbidity and mortality. 

Due to its relative rarity, there is little guidance on the management of Bouveret syndrome or a standardized approach. Endoscopic, laparoscopic, and open approaches can be diagnostic and interventional as in the case outlined in this report. In addition, this case demonstrates that stone migration can complicate the management of Bouveret syndrome and necessitate more extensive surgical intervention than originally anticipated. Surgeons should be aware of this in order to plan their surgical approach and consent accordingly.
